# Degree of Early Estrogen Response Predict Survival after Endocrine Therapy in Primary and Metastatic ER-Positive Breast Cancer

**DOI:** 10.3390/cancers12123557

**Published:** 2020-11-28

**Authors:** Masanori Oshi, Yoshihisa Tokumaru, Fernando A. Angarita, Li Yan, Ryusei Matsuyama, Itaru Endo, Kazuaki Takabe

**Affiliations:** 1 Department of Surgical Oncology, Roswell Park Comprehensive Cancer Center, Buffalo, New York, NY 14263, USA; masa1101oshi@gmail.com (M.O.); Yoshihisa.Tokumaru@roswellpark.org (Y.T.); fernando.angaritacelis@roswellpark.org (F.A.A.); 2 Department of Gastroenterological Surgery, Yokohama City University Graduate School of Medicine, Yokohama 236-0004, Japan; ryusei@yokohama-cu.ac.jp (R.M.); endoit@yokohama-cu.ac.jp (I.E.); 3 Department of Surgical Oncology, Graduate School of Medicine, Gifu University, 1-1 Yanagido, Gifu 501-1194, Japan; 4 Department of Biostatistics & Bioinformatics, Roswell Park Comprehensive Cancer Center, Buffalo, New York, NY 14263, USA; li.yan@roswellpark.org; 5 Division of Digestive and General Surgery, Niigata University Graduate School of Medical and Dental Sciences, Niigata 951-8520, Japan; 6 Department of Breast Surgery, School of Medicine, Fukushima Medical University, Fukushima 960-1295, Japan; 7 Department of Surgery, Jacobs School of Medicine and Biomedical Sciences, State University of New York, Buffalo, New York, NY 14263, USA; 8 Department of Breast Surgery and Oncology, Tokyo Medical University, Tokyo 160-8402, Japan

**Keywords:** biomarker, GSEA, GSVA, estrogen response, endocrine, treatment response, survival, ER

## Abstract

**Simple Summary:**

Over 70% of breast cancer patients in the US have estrogen receptor (ER)-positive tumors. A better predictive biomarker of endocrine therapy would be useful to improve patient compliance. We found that estrogen response early score generated by a GSEA algorithm was significantly associated with immune cell infiltrations, patient survival, and endocrine therapy response in ER-positive breast cancer. Furthermore, the score may be a useful tool to predict response to endocrine therapy in both primary and metastatic breast cancer compared to ER gene expression. This study demonstrates that the estrogen response early score may be a useful prognostic and predicative biomarker in patients with either primary or metastatic breast cancer.

**Abstract:**

Endocrine therapy is the gold-standard treatment for ER-positive/HER2-negative breast cancer. Although its clear benefit, patient compliance is poor (50–80%) due to its long administration period and adverse effects. Therefore, a predictive biomarker that can predict whether endocrine therapy is truly beneficial may improve patient compliance. In this study, we use estrogen response early gene sets of gene set enrichment assay algorithm as the score. We hypothesize that the score could predict the response to endocrine therapy and survival of breast cancer patients. A total of 6549 breast cancer from multiple patient cohorts were analyzed. The score was highest in ER-positive/HER2-negative compared to the other subtypes. Earlier AJCC stage, as well as lower Nottingham pathological grade, were associated with a high score. Low score tumors enriched only allograft rejection gene set, and was significantly infiltrated with immune cells, and high cytolytic activity score. A low score was significantly associated with a worse response to endocrine therapy and worse survival in both primary and metastatic breast cancer patients. The hazard ratio was double that of *ESR1* expression. In conclusion, the estrogen response early score predicts response to endocrine therapy and is associated with survival in primary and metastatic breast cancer.

## 1. Introduction

Estrogen receptor (ER)-positive breast cancers constitute the most common subtype representing approximately 75% of breast cancer [1]. Patients with ER-positive breast cancers have relatively better survival compared to those with the other subtypes. The rationale for improved survival is because these patients respond to endocrine therapy, namely, tamoxifen, aromatase inhibitors, and fulvestrant. Endocrine therapy is used in several settings, including neoadjuvant, adjuvant, as well as metastatic ER-positive breast cancer. Adjuvant endocrine therapy reduces the risk of recurrence by approximately 50% and improves survival [2,3]. Although the side effects of endocrine therapy are mainly post-menopausal symptoms and are considered mild compared to cytotoxic chemotherapy, they can be bothersome for some patients to the point that 20–50% of the patients are unable to complete the full 5-year course of treatment [4,5]. To this end, a predictive biomarker of endocrine therapy could improve patient compliance by clarifying individual patient’s benefit to treatment. Furthermore, such biomarker may help surgeons to select patients for neoadjuvant endocrine therapy who are more likely to benefit from that approach.

Drug response is modulated by complex interactions of multiple genes. Therefore, analyzing a single gene will not capture the global kinetic of multiple genes involved in the process like gene expression scoring would do [6,7]. We have previously reported the usefulness of scoring using signaling gene sets to understand the clinical relevance of several pathways in cancer [8,9,10,11]. In this study, we used the “hallmark estrogen response early gene set”, which contains 200 genes, in Molecular Signatures Database (MSigDB) gene set collections of the gene set variation analysis (GSVA) algorithm [12] as the score to measure estrogen response pathway activity of breast cancer. We also examined its association with various pathological and clinical features of breast cancer. We hypothesized that an elevated estrogen response early score is associated with a better response to endocrine therapy and better survival in both primary and metastatic ER-positive breast cancer.

## 2. Results

### 2.1. Estrogen Response Early Score Was Significantly Higher in the Estrogen Receptor (ER)-Positive Breast Cancer Compared with the Other Subtype, and Correlated with ESR1 but Not with ESR2 Expressions

We used the estrogen response score based on gene set variation analysis (GSVA) score for the MSigDB hallmark estrogen response early gene set (Table S1), which contains 200 genes that are involved in the estrogen response signaling, as we previously reported for the other scores [8,9,10,11]. The top one-third of the score within each cohort was used as the cut-off to divide patients into high- and low-score groups.

**Figure 1:**
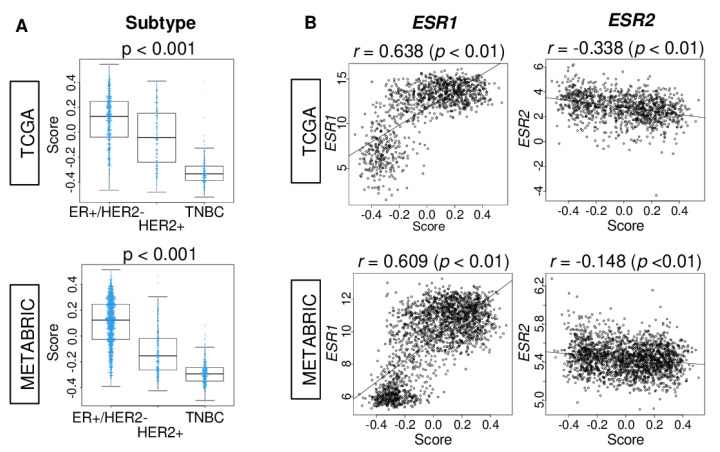
The association of the estrogen response early score with breast cancer subtype and estrogen receptor (ER) gene expressions in the TCGA and METABRIC cohorts. (**A**) Boxplots of the comparison of estrogen response early score by subtype; ER-positive/human epidermal receptor (HER2)-negative, HER2-positive, and triple-negative breast cancer (TNBC) in the TCGA and METABRIC cohorts. *p*-value was calculated by one-way ANOVA test. (**B**) Correlation plots of the score with the expression of the *ESR1* and *ESR2* genes in the TCGA and METABRIC cohorts. The *p*-value was calculated by Spearman’s rank correlation coefficient.

We first studied the association of the estrogen response score with the ER status of breast cancer. We examined the association of the score with different breast cancer subtypes. The estrogen response early score in ER-positive/human epidermal receptor 2 (HER2)-negative breast cancer, determined by immunohistochemistry, was highest compared to the other subtypes in the TCGA and METABRIC cohorts (Figure 1A; both *p* < 0.001). Given that *ESR1* and *ESR2* encode for ER-α and ER-β, we investigated the correlation of the estrogen response early score with the expression of ER-α (*ESR1*) and ER-β (*ESR2*) genes in large breast cancer cohorts. We found that the score was moderately correlated with the expression of *ESR1*, but not with *ESR2* genes in both TCGA and METABRIC cohorts (Figure 1B; *ESR1*, *r* = 0.638, *p* < 0.01; *r* = 0.609, *p* < 0.01, *ESR2*, *r* = −0.338, *p* < 0.01; *r* = −0.148, *p* < 0.01, respectively).

### 2.2. High Estrogen Response Early Score Was Significantly Associated with Less Tumor Aggressiveness and Better Survival in ER-Positive/HER2-Negative Breast Cancer

**Figure 2:**
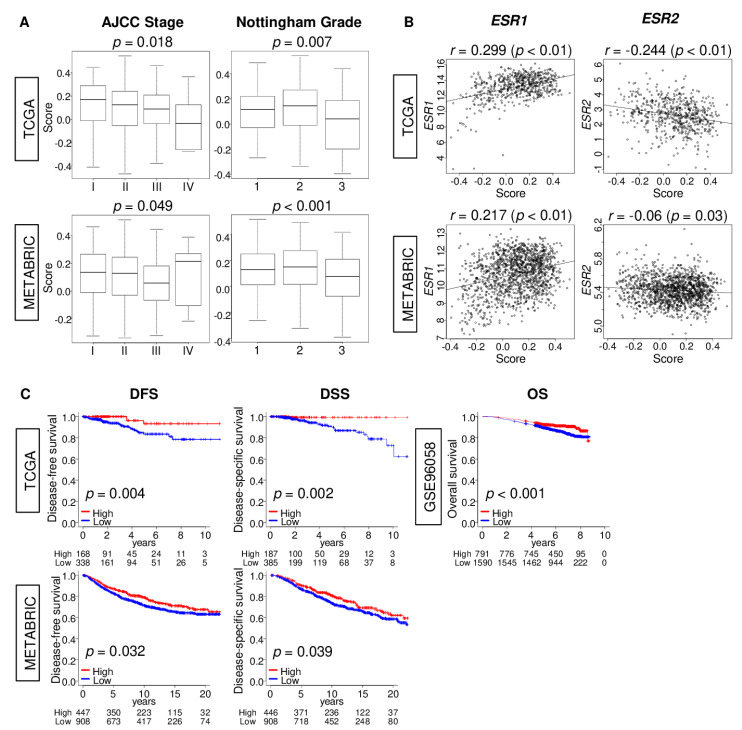
Association of the estrogen response early score with clinical features in estrogen receptor (ER)-positive/human epidermal receptor (HER2)-negative breast cancer. (**A**) Boxplots of the comparison of the estrogen response score by the American Joint Committee on Cancer (AJCC) staging (Stage I-IV), and Nottingham pathological grade in the TCGA and METABRIC cohorts. *p*-value was calculated by one-way ANOVA test. (**B**) Correlation plots of the score with *ESR1* and *ESR2* gene expressions in ER-positive/HER2-negative breast cancer in both cohorts. *p*-value was calculated by Spearman’s rank correlation coefficient. (**C**) Kaplan-Meier survival curve with a log-rank *p*-value between low- (blue) and high- (red) score in ER-positive/HER2-negative breast cancer with disease-free survival (DFS), disease-specific survival (DSS) in the TCGA and METABRIC cohorts, and overall survival (OS) in the GSE96058 cohort. The top one-third was defined as the high score group within each cohort.

Given the better clinical outcome, we hypothesized that ER-positive/HER2-negative have better cancer biology in addition to the response to endocrine therapy compared to other subtypes. To test this hypothesis, we investigated the association of estrogen response early score with clinical features, including the American Joint Committee on Cancer (AJCC) stage and Nottingham pathological grade at the time of diagnosis prior to any treatment. We found that a high estrogen response early score was significantly associated with earlier stage, as well as lower pathological grade consistently in both TCGA and METABRIC cohorts (Figure 2A; stage *p* = 0.018, *p* = 0.049; grade *p* = 0.007, *p* < 0.001, respectively).The score showed no correlation with either *ESR1* or *ESR2* gene expressions in ER-positive/HER2-negative breast cancer in the TCGA and METABRIC cohorts (Figure 2B; *ESR1*, *r* = 0.299, *r* = 0.217; *ESR2*, *r* = −0.244, *r* = −0.06). A high score was significantly associated with better disease-free survival (DFS) (*p* = 0.004) and disease-specific survival (DSS) (Figure 2C; *p* = 0.002) in ER-positive/HER2-negative subtype in the TCGA cohort. These results were validated in the METABRIC cohort (Figure 2C; DFS; *p* = 0.032, DSS; *p* = 0.039), as well as the GSE96058 cohort (Figure 2C; overall survival (OS); *p* < 0.001) cohorts. These findings suggest that high estrogen response early score was associated with less aggressive ER-positive/HER2-negative breast cancer, which did not correlate with ER gene expression.

### 2.3. Allograft Rejection Was the Only Gene Set that Enriched to Low Estrogen Response Early Score Breast Cancer in METABRIC and GSE96058 Cohorts

**Figure 3:**
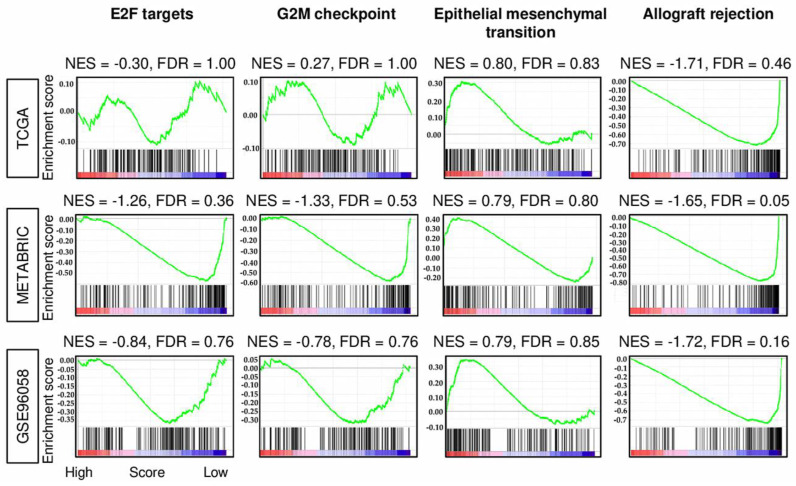
Gene set enrichment analysis (GSEA) of estrogen response early score in ER-positive/HER2-negative breast cancer. GSEA plots of the comparison between low and high estrogen response gene with normalized enrichment score (NES) and false discovery rate (FDR) in the TCGA, METABRIC, and GSE96058 cohorts. FDR < 0.25 was statistically significant in the analysis, as recommended by GSEA software.

To reveal the functional characteristics of the estrogen response early score where the low score associated with aggressive cancer biology, we performed a pathway analysis using the Gene Set Enrichment Analysis (GSEA) with Hallmark gene sets in ER-positive/Her2-negative breast cancer. There were no cancer aggressiveness related gene sets, such as proliferation-related or metastasis-related that significantly enriched to either high or low score group (Figure 3). The exception was allograft rejection, which is an immune response-related gene set [13], enriched to the low score, which was associated with aggressive features. These results suggest that the hallmark pathways involved in the high score group that was associated with better clinical features and survival were the estrogen response pathway alone.

### 2.4. Low Estrogen Response Early Score Was Associated with High Infiltration of Both Anti- and Pro- Cancer Immune Cells as Well as B Cells in ER-Positive/HER2-Negative Breast Cancer

**Figure 4:**
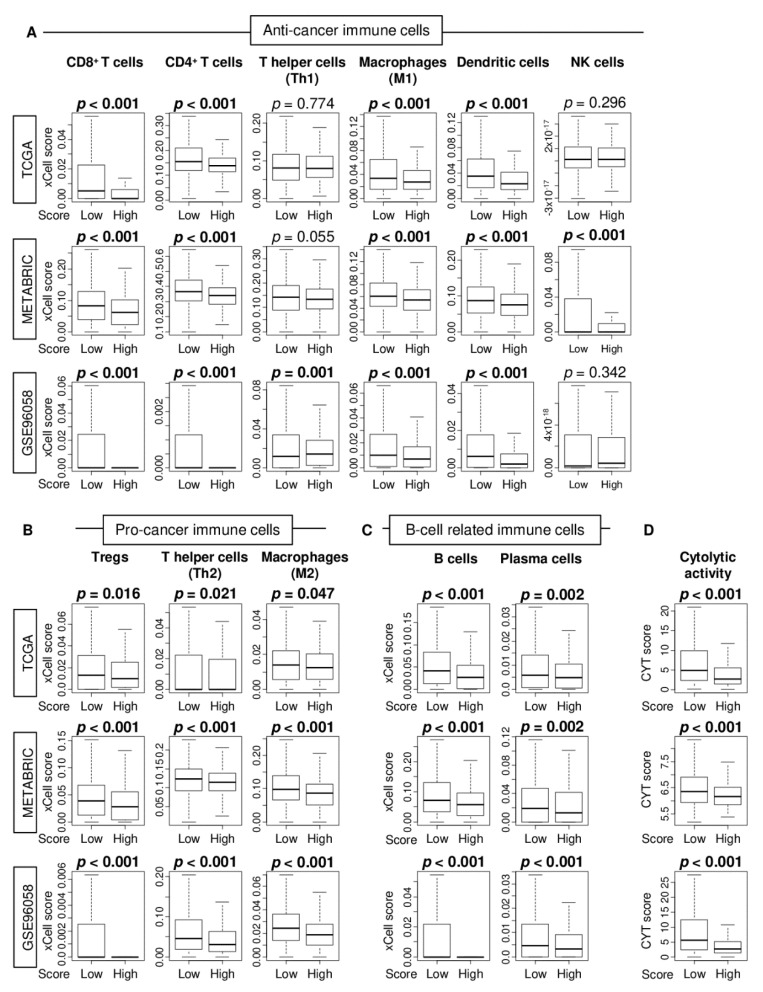
Association of the estrogen response early score and infiltrating immune cells in the estrogen receptor (ER)-positive/human epidermal receptor (HER2)-negative breast cancer. Boxplots of the comparison with a fraction of (**A**) anti-cancer immune cells (CD8^+^ T cells, CD4^+^ T cells, T helper type 1 (Th1) cells, M1 macrophages, dendritic cells, and NK cells), (**B**) pro-cancer immune cells (regulatory T cells [Tregs], T helper type 2 (Th2) cells, and M2 macrophages), and (**C**) B cells and plasma cells, as well as (**D**) the cytolytic activity score by low and high estrogen response early score group in the TCGA, METABRIC, and GSE96058 cohorts. *p*-value was calculated by one-way ANOVA test.

Given the GSEA result that low score ER-positive/HER2-negative breast cancers were enriched with immune-related gene set, it was of interest to study the association of the estrogen response score and tumor immune microenvironment (TIME) in ER-positive/HER2-negative breast cancer. The fraction of immune cells in TIME was estimated using the xCell algorithm between low and high estrogen response early score group in the TCGA, METABRIC, and GSE96058 cohorts. We found that low estrogen response early score tumors were significantly infiltrated with anti-cancer immune cells, including CD8^+^ T cells, CD4^+^ T cells, M1 macrophages, dendritic cells, and NK cells, as well as pro-cancer immune cells, including regulatory T cells (Tregs), T helper type 2 (Th2) cells, and M2 macrophages consistently in all three cohorts (Figure 4A,B). Low score tumors were also infiltrated with B cells and plasma cells consistently in all three cohorts (Figure 4C). T helper type 1 (Th1) cells significantly infiltrated in low score tumors in GSE96058 cohort alone. Furthermore, the low score group was significantly associated with enhanced cytolytic activity consistently in all three cohorts (Figure 4D). These results suggest that low estrogen response score was associated with high infiltration of both anti- and pro-cancer immune cells, which result in enhanced cytolytic activity in TIME, which is in agreement with GSEA results.

### 2.5. High Estrogen Response Early Score Was Significantly Associated with Better Response to Endocrine Therapy in ER-Positive Breast Cancer More Strongly than ESR1 Expression

**Figure 5:**
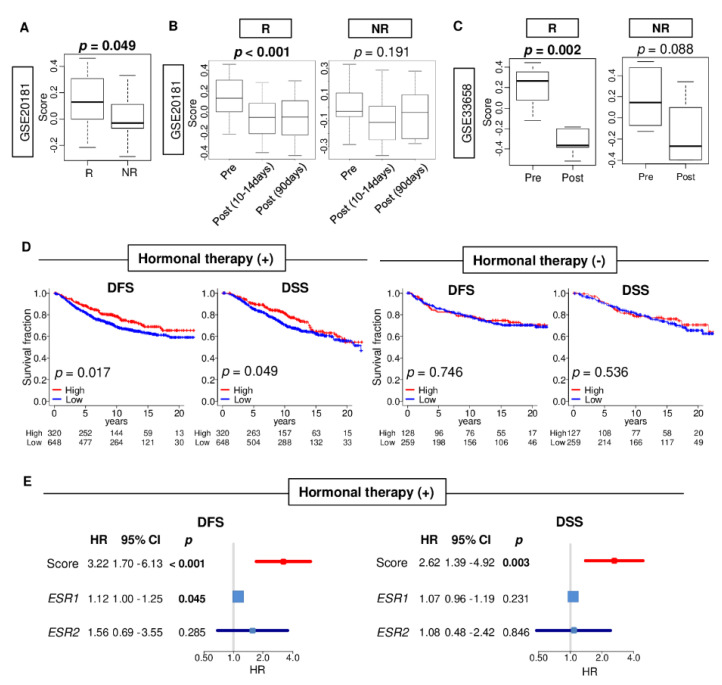
Association of the estrogen response early score with treatment response to endocrine therapy in estrogen receptor (ER)-positive breast cancer. (**A**) A boxplot of the comparison of the score between response (R) and non-response (NR) group in the GSE20181 cohort (*n* = 176) that received letrozole. (**B**) Boxplots comparing the score between pre-treatment, post- (10–14 days) and post- (90 days) treatment tumors in the GSE20181 cohort. (**C**) Boxplots of the comparison of the score between pre- and post-treatment tumors in the response group and non-response group in the GSE33658 cohort that underwent anastrozole and fulvestrant (*n* = 22). *p*-value was calculated by one-way ANOVA test. (**D**) Kaplan-Meier with log-rank *p*-value between low (blue) and high (red) score with disease-free survival (DFS) and disease-specific survival (DSS) in the treatment group and non-treatment group in the METABRIC cohort. The top one-third was defined as the high score group within the cohort. (**E**) Forrest plots of the estrogen response early score, *ESR1* and *ESR2* expression with DFS and DSS in an endocrine therapy group in the METABRIC cohort. The *p*-value was calculated by Cox regulation model. HR; hazard ratio, CI; confidence interval.

Given that the estrogen response early score was configured by genes involved in endocrine treatment response [14,15,16,17], we hypothesized that patients with a high estrogen response early score have a better response to endocrine therapy. To test this hypothesis, we investigated the association of the score between good response group (R) and non-response group (NR) to endocrine therapy with letrozole in GSE20181 cohort (Figure 5A, *n* = 176). Estrogen response early score was significantly higher in the good response group (R) compared to the non-response group (NR) (*p* = 0.049). Furthermore, when the score was compared between pre- and post-treatment tumors, the score was significantly decreased in both early- and long-term after treatment in the good response group (R), but not in the non-response group (NR) in GSE20181 cohorts (Figure 5B; *p* < 0.001 and *p* = 0.191, respectively). The result was validated in the GSE33658 cohort (*n* = 22) in which anastrozole and fulvestrant were administered (Figure 5C; *p* = 0.002 and 0.088, respectively), although there was a trend in NR group.

Next, patients with a low or high score were compared in the endocrine treatment group and the non-endocrine treatment group in the METABRIC cohort. Patients with high scores ER-positive/HER2-negative breast cancer have significantly better DFS and DSS in treatment group, but not in the non-treatment group in the METABRIC cohort (Figure 5D). These findings suggest that the score may predict the response to endocrine therapy. This finding is in agreement with the results that high score was significantly associated with better survival in ER-positive breast cancer patients, with endocrine therapy group. Furthermore, the hazard ratio of DFS and DSS were compared between estrogen response score, *ESR1* and *ESR2* expressions. In this case we found that the score showed the largest hazard ratio (Figure 5E; DFS; HR = 3.23 (*p* < 0.001), 1.12 (*p* = 0.045), 1.56 (*p* = 0.285), DSS; HR = 2.62 (*p* = 0.003), 1.07 (*p* = 0.231), 1.08 (*p* = 0.846), respectively). These results suggest that estrogen response early score was associated with a better response to endocrine therapy in ER-positive breast cancer. Additionally, it was more strongly predictive relative to *ESR1* expression.

### 2.6. High Estrogen Response Early Score Metastatic Tumors Were Significantly Associated with Better Survival in Metastatic Breast Cancer More Strongly than ESR1 Expression

**Figure 6:**
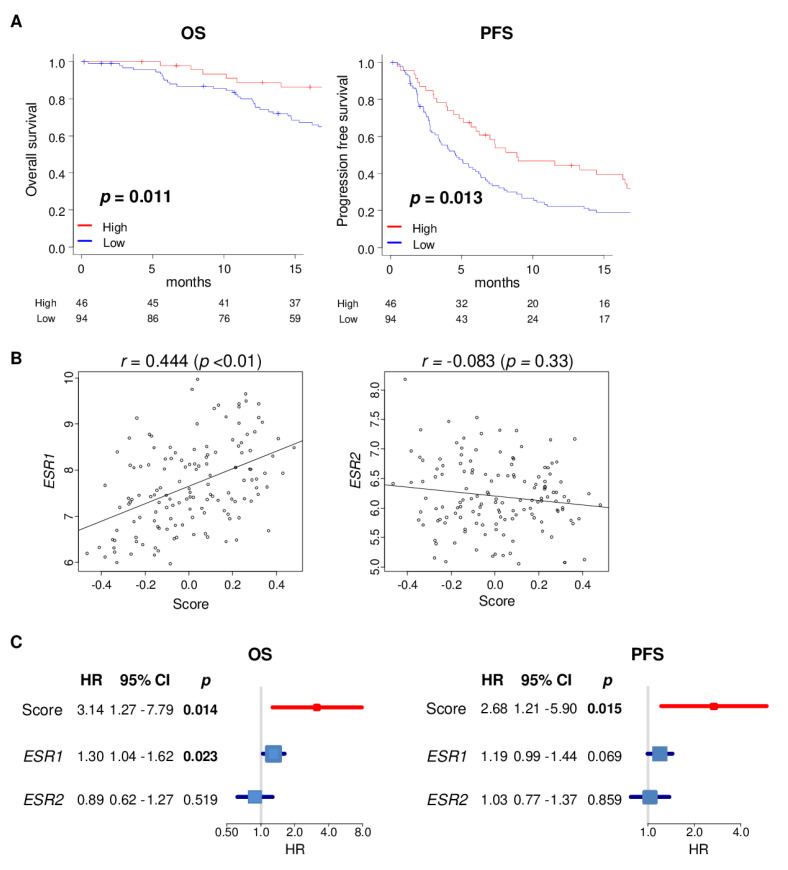
Association of the estrogen response early scores with patient survival in metastatic breast cancer in the GSE124647 cohort (*n* = 140). (**A**) Kaplan-Meier with log-rank *p*-value between low (blue) and high (red) score with overall survival (OS) and progression-free survival (PFS). The top one-third was defined as the high score group within the cohort. (**B**) Correlation plots of the score with the expression of the *ESR1* or *ESR2* genes in the GSE124647 cohort. *P*-value was calculated by Spearman’s rank correlation coefficient. (**C**) Forrest plots of the estrogen response early score, *ESR1* and *ESR2* expression with OS and PFS. *P*-value was calculated by cox regulation model. HR; hazard ratio, CI; confidence interval.

Because high score tumors have better biology, and they respond to endocrine therapy in ER-positive tumors, we expected that patients with high estrogen response early score have better survival compared with a low score in metastatic breast cancer. Using the GSE124647 metastatic breast cancer cohort (*n* = 140) that has both clinical outcome and gene expression data, we found that a high score was significantly associated with better OS, as well as PFS in metastatic breast cancer patients (Figure 6A; *p* = 0.011, and 0.013, respectively). The score demonstrated only a weak correlation with *ESR1* expression and no correlation with *ESR2* in this metastatic cohort (Figure 6B, *r* = 0.444 and *r* = −0.083, respectively). Furthermore, we found that the hazard ratio of the score was significantly stronger than *ESR1* or *ESR2* expression in both OS and PFS (Figure 6C; OS; HR = 3.14 (*p* = 0.014), 1.30 (*p* = 0.023), 0.89 (*p* = 0.519), PFS; 2.68 (*p* = 0.015), 1.19 (*p* = 0.069), 1.03 (*p* = 0.859), respectively). These results suggest that the estrogen response early score was associated with better OS and PFS in metastatic breast cancer, and it was a stronger prognostic biomarker than *ESR1* expression. Although a very small sample size of each metastatic tumor may lack the power to achieve statistical significance, high score tumors tended to be associated with better survival (Figure S1). This result supports the notion that the score is associated with survival and not with the location of the metastatic tumor.

## 3. Discussion

In this study, we studied the clinical relevance of the early estrogen response using the estrogen response early score, which was determined by “Hallmark Estrogen response early gene set” in GSVA. By analyzing multiple large patient cohorts with transcriptomes, we found that estrogen response early scores only moderately correlated with the expression of *ESR1* in the whole breast cancer cohort. However, there was no correlation between ER-positive/HER2-negative breast cancer. Earlier AJCC stage and lower pathological grade were significantly associated with a high score, which was significantly associated with better survival among ER-positive/HER2-negative breast cancer consistently in all three cohorts. Allograft rejection, one of the immune response-related gene sets alone, and none of the cell proliferation-related (E2F targets and G2M checkpoint) or metastasis-related (epithelial mesenchymal transition) gene sets were enriched in the low estrogen response score group in two of the three cohorts. A low estrogen response score tumor was significantly infiltrated with both anti- and pro-cancer immune cells, as well as B cells, including CD8^+^ T cells, CD4^+^ T cells, M1 macrophages, dendritic cells, regulatory T cells, Th2 cells, M2 macrophages, and plasma cells, and associated with enhanced cytolytic activity score. Patients who responded to endocrine therapy had higher scores than non-responders, which decreased significantly after treatment by three months. However, this was not the case in non-responders. The high score group was significantly associated with better prognosis in patients who received endocrine therapy, whereas there was no difference in survival in patients without endocrine therapy. The hazard ratio of the score for both DFS and DSS was significantly higher than *ESR1* or *ESR2* expression. Interestingly, metastatic breast cancer patients with high scores were associated with better PFS and OS compared to low score group. The score showed no correlation with *ESR1* expression, and hazard ratio of the score was more than two-fold of *ESR1* expression.

There are two subtypes of ER, namely, ERα and ERβ, encoded by *ESR1* and *ESR2*, respectively. Endocrine therapy is widely used for breast cancer in the neoadjuvant, adjuvant, and metastatic setting, prolonging overall, and recurrence-free survival [2,18]. Whilst endocrine therapy is initially effective for ER-positive breast cancer patients, many develop resistance; thus, the *ESR1* expression level is not an effective predictor for endocrine therapy. In this study, we found that the estrogen response early score is a stronger prognostic and predictive biomarker than *ESR1* expression, not only in primary, but also in metastatic breast cancer.

The lack of enrichment of the pro-cancer pathways (cell proliferation-related and metastasis-related gene sets) in the low score group with poor prognosis is in agreement with the notion that ER-positive breast cancer is dependent on estrogen response for its aggressiveness that translates into survival. Further, the fact that allograft rejection gene sets enriched to the low score group, as well as higher infiltration of multiple immune cells and elevation of cytolytic activity in the low score ER-positive subtype, align with the known trend that triple-negative breast cancer is more immunogenic although more aggressive than ER-positive subtype. Taken together with our result that neither *ESR1* nor *ESR2* expression did not correlate well with estrogen response within ER-positive subtype, we believe that the score may be a predictor than *ESR1*. Several prognostic scores have been developed in ER-positive patients using gene expression and clinical information, such as 21-gene Oncotype DX, 70-gene MammaPrint, 50-gene PAM50, 12-gene EndoPredict, and Breast Cancer index [19,20]. However, none of them have predicted response to endocrine treatment to date. Our results demonstrate the association of the score with treatment response and survival, indicating the possibility of using it as a predictive and prognostic biomarker for the response to endocrine therapy.

Endocrine therapy is also used for ER-positive metastatic breast cancer. These patients usually have a median five-year survival of 27%. This is partly because those ER-positive tumors have developed resistance to endocrine therapy. To that end, accurate predictors for endocrine therapy response in metastatic breast cancer are even more helpful for appropriate patient selection in a population as side effects need to be minimized. Interestingly, the fact that high estrogen response early score was associated with better prognosis in metastatic tumors and the hazard ratio of the score was higher than that of *ESR1* expression indicated that the score may be useful as a predictive biomarker for metastatic breast cancer.

Many factors contribute to non-adherence or discontinuation of long-term endocrine therapy. It is not uncommon that patients state a fear of the side effects [21]. Other patients discontinue the medication because they underestimate the benefit of adjuvant treatment as they feel “cancer-free”. The cost and affordability of prescription medications is a major concern for many, and some patients may have difficulty remembering to take the drugs regularly. This score will clarify the benefit of adjuvant endocrine therapy to each individual patient. We hypothesize that the score will improve patient compliance and the prognosis of ER-positive/HER2-negative breast cancer patients. Furthermore, it may have a clinical utility to be used for patient selection for endocrine therapy, and to select patients who can benefit from treatment by the score can also help reduce the number of patients who suffer from adverse effects with ineffective treatment.

Although this current study exclusively analyzed the transcriptome of patient cohorts, this by no means intended to undermine the importance of experiments using preclinical models for a better understanding of cancer biology. Mercatali et al. reported the utility of an in vitro preclinical model of TNBC and osteoclasts co-cultured with peripheral blood monocytes [22]. Our group established an improved syngeneic orthotopic murine model [23,24,25,26] and used to elucidate the mechanisms in immunological [27], pharmacological [28,29], and surgical [30,31,32] topics, as well as the mechanism how S1P link inflammation and breast cancer progression [33]. We reported the improved technique to generate patient-derived xenograft (PDX) [34,35] and reported the utility of the novel breast cancer brain metastatic PDX model [36]. Thus, preclinical models are essential tools to elucidate cancer biology. With that said, we are also aware that no model can perfectly replicate human cancer. For example, how much syngeneic murine cells model human cancer cells are unknown, and PDX model ignores the tumor immune microenvironment, since it utilizes immunodeficient mice. To this end, we strongly believe that future cancer research will require integration of multiple modalities, such as in vitro and in vivo models for molecular biological and pathological analyses, but also in silico analyses to snapshot the phenomenon happening in real human cancer.

With the recent advances in RNA-sequencing and gene expression microarray technologies, the cost has decreased significantly from 2.7 billion US Dollars for the Human Genome Project to sequence the entire genome in 2005 to several hundred US Dollars to RNA-sequence all the genes today. Using gene expression profile of all the genes, our score predicts the likelihood of response to endocrine therapy, at the same time such profile allows us to grasp the tumor’s biology and its tumor microenvironment as we have reported from expressed genes [37,38,39], immune cells [40,41,42], stromal cells [43,44], and pathways [45,46] stand point. To this end, RNA-sequence of tumors may become a routine in the near future. Compared with the costs of the other tests currently in the market (several thousands of US Dollars), this may be a justifiable investment. Although some of the genes may be associated stronger to endocrine therapy response among the 200 genes included in the score, we do not recommend to quantify the expressions of these selected genes because generating a specific panel for them most likely cost higher than several hundred US Dollars, as well as the utility of the gene expression profile of all the genes.

This study is not free from limitations. Although we utilized multiple large patient cohorts, this is a retrospective study with publicly available cohorts of previous studies. The cohorts we examined vary from each other by patient background and clinical characteristics. TCGA cohort is known to lack co-morbidities and therapeutic intervention data. Furthermore, we could not investigate the association of the score with the effects of other treatments, due to lack of other treatment data, such as adjuvant chemotherapy and radiation therapy. Finally, this study did not assess the biological mechanisms that underlie its clinical findings.

## 4. Materials and Methods

### 4.1. Clinical and Transcriptomic Data Collection for Breast Cancer Patients

We obtained transcriptomic and clinical data of 1069 female patients who had a histopathological diagnosis of breast cancer in The Cancer Genome Atlas Breast Cancer (TCGA-BRCA) cohort [47]. We also obtained the data of 1904 patients in the Molecular Taxonomy of Breast Cancer International Consortium (METABRIC) cohort [48,49] through cBioPortal [50] with these similar features. These data were obtained from studies including Miller et al. (GSE20181; *n* = 176) [51], Massarweh et al. (GSE33658; *n* = 22) [52], Sinn et al. (GSE124647; *n* = 140) [53], and Curtis et al. (GSE96058; *n* = 3234) [54], through the Gene Expression Omnibus (GEO) repository. Clinical and pathological features of each cohort are demonstrated in Supplemental Tables S2–S10. All the cohorts used in this study, TCGA, METABRIC and GEO data sets, are de-identified publicly available databases. Given that the patient data are de-identified, and that it is in public domain, the ethics committee of our institution waived Institutional Review Board (IRB) approval.

### 4.2. Estimate the Estrogen Response Early Score

“Hallmark estrogen response early” gene set (Table S1) in the MSigDB Hallmark collection of Gene set variation analysis (GSVA) score of the [55] was used to measure the estrogen response early pathway score. GSVA Bioconductor package (version 3.10) was used.

### 4.3. Gene Set Enrichment Analysis

Gene et Enrichment Analysis (GSEA) [13] was used to identify the significant Hallmark pathways enriched in the high score group, as we previously reported [38,56,57,58]. The statistically significant pathways enriched in the high score group were defined by a false discovery rate a less than 0.25, as recommended by GSEA software.

### 4.4. Tumor Immune Microenvironment Analysis

xCell algorithm [59] was used to estimate the fraction of immune cells in the tumor microenvironment using transcriptomic data of the whole tumor, as we previously reported [37,42,44,60,61,62].

### 4.5. Other Statistical Analyses

All statistical tests were performed using R software (https://www.r-project.org/version 4.0.1, R Project for Statistical Computing). We chose the top one-third as a cut-off to select hormonal therapy-sensitive patient population, based on the results from IMPACT trial that 22–46% of the ER-positive locally advanced breast cancer responded to neoadjuvant hormonal therapy [63]. All plots were constructed using R software and Microsoft Excel (version 16, Redmond, WA, USA) for Windows. One-way analysis of variance (ANOVA) or Fisher’s exact tests were used to compare group means. The survival differences between groups were analyzed using Kaplan-Meier curves with a log-rank test. Cox proportional hazard analyses were used to estimate HR, 95% CI, and *p* value. *p*-values were considered as significant when less than 0.05.

## 5. Conclusions

The estrogen response early score have potential as both a prognostic and predictive biomarker for endocrine therapy in both primary and metastatic ER-positive breast cancer with hazard ratio double of that of *ESR1* expression.

## Supplementary Materials

The following are available online at https://www.mdpi.com/2072-6694/12/12/3557/s1, Table S1: Symbols and names of genes that constitute the estrogen response early gene sets, Table S2: Clinical and pathological features of each subtype in the TCGA cohort, Table S3: Comparison of clinical and pathological features between low and high estrogen response early score with ER-positive/HER2-negative breast cancer in the TCGA cohort, Table S4: Clinical and pathological features of each subtype in the METABRIC cohort, Table S5: Comparison of clinical and pathological features between low and high estrogen response early score with ER-positive/HER2-negative breast cancer in the METABRIC cohort, Table S6: Clinical and pathological features of each subtype in the GSE96058 cohort, Table S7: Comparison of clinical and pathological features between low and high estrogen response early score with ER-positive/HER2-negative breast cancer in the GSE96058 cohort, Table S8: Comparison of metastatic regions between low and high estrogen response early score in the GSE124647 cohort, Table S9: Clinical and pathological status of samples in the GSE20181 cohort, Table S10: Clinical and pathological status of samples in the GSE33658 cohort, Figure S1: Association of the estrogen response early score with patient survival in several metastatic tumor in the GSE124647 cohort (*n* = 140).

## Abbreviations

AJCC, American Joint Committee on Cancer; DFS, disease-free survival; DSS, disease-specific survival; ER, estrogen receptor; FDR, false discovery rate; HER2, human epidermal growth factor receptor 2; METABRIC, Molecular Taxonomy of Breast Cancer International Consortium; NES, normalized enrichment score; OS, overall survival; pCR, pathological complete response; TCGA, The Cancer Genome Atlas; TNBC, triple-negative breast cancer.
