# Supplementary material for: Degree of Early Estrogen Response Predict Survival after Endocrine Therapy in Primary and Metastatic ER-Positive Breast Cancer
Source: Cancers (Basel). 2020 Nov 28;12(12):3557. doi: 10.3390/cancers12123557 (PMC7760577; doi:10.3390/cancers12123557)
Supplement: Supplementary file 1 [file cancers-12-03557-s001.pdf]

Supplementary Materials: Degree of Early Estrogen Response Predict Survival after Endocrine Therapy in Primary and Metastatic ER-Positive Breast Cancer

Masanori Oshi, Yoshihisa Tokumaru, Fernando A. Angarita, Li Yan, Ryusei Matsuyama, Itaru Endo and Kazuaki Takabe

Table S1. Symbols and names of genes that constitute the estrogen response early gene sets.

Gene Symbol	Gene Name
ABAT	4-aminobutyrate aminotransferase
ABCA3	ATP binding cassette subfamily A member 3
ABHD2	abhydrolase domain containing 2, acylglycerol lipase
ABLIM1	actin binding LIM protein 1
ADCY1	adenylate cyclase 1
ADCY9	adenylate cyclase 9
ADD3	adducin 3
AFF1	AF4/FMR2 family member 1
AKAP1	A-kinase anchoring protein 1
ALDH3B1	aldehyde dehydrogenase 3 family member B1
AMFR	autocrine motility factor receptor
ANXA9	annexin A9
AQP3	aquaporin 3 (Gill blood group)
AR	androgen receptor
AREG	amphiregulin
ARL3	ADP ribosylation factor like GTPase 3
ASB13	ankyrin repeat and SOCS box containing 13
B4GALT1	beta-1,4-galactosyltransferase 1
BAG1	BAG cochaperone 1
BCL11B	BAF chromatin remodeling complex subunit BCL11B
BCL2	BCL2 apoptosis regulator
BHLHE40	basic helix-loop-helix family member e40
BLVRB	biliverdin reductase B
CA12	carbonic anhydrase 12
CALB2	calbindin 2
CALCR	calcitonin receptor
CANT1	calcium activated nucleotidase 1
CBFA2T3	CBFA2/RUNX1 partner transcriptional co-repressor 3
CCND1	cyclin D1
CD44	CD44 molecule
CELSR1	cadherin EGF LAG seven-pass G-type receptor 1
CELSR2	cadherin EGF LAG seven-pass G-type receptor 2
CHPT1	choline phosphotransferase 1
CISH	cytokine inducible SH2 containing protein
CLDN7	claudin 7
CLIC3	chloride intracellular channel 3
CXCL12	C-X-C motif chemokine ligand 12
CYP26B1	cytochrome P450 family 26 subfamily B member 1
DEPTOR	DEP domain containing MTOR interacting protein
DHCR7	7-dehydrocholesterol reductase

DHRS2	dehydrogenase/reductase 2
DHRS3	dehydrogenase/reductase 3
DLC1	DLC1 Rho GTPase activating protein
DYNLT3	dynein light chain Tctex-type 3
EGR3	early growth response 3
ELF1	E74 like ETS transcription factor 1
ELF3	E74 like ETS transcription factor 3
ELOVL2	ELOVL fatty acid elongase 2
ELOVL5	ELOVL fatty acid elongase 5
ENDOD1	endonuclease domain containing 1
ESRP2	epithelial splicing regulatory protein 2
FAIM3	Fc fragment of IgM receptor
FAM102A	family with sequence similarity 102 member A
FAM134B	reticulophagy regulator 1
FAM63A	MINDY lysine 48 deubiquitinase 1
FARP1	FERM, ARH/RhoGEF and pleckstrin domain protein 1
FASN	fatty acid synthase
FDFT1	farnesyl-diphosphate farnesyltransferase 1
FHL2	four and a half LIM domains 2
FKBP4	FKBP prolyl isomerase 4
FKBP5	FKBP prolyl isomerase 5
FLNB	filamin B
FOS	Fos proto-oncogene, AP-1 transcription factor subunit
FOXC1	forkhead box C1
FRK	fyn related Src family tyrosine kinase
GAB2	GRB2 associated binding protein 2
GFRA1	GDNF family receptor alpha 1
GJA1	gap junction protein alpha 1
GLA	galactosidase alpha
GREB1	growth regulating estrogen receptor binding 1
HES1	hes family bHLH transcription factor 1
HR	HR lysine demethylase and nuclear receptor corepressor
HSPB8	heat shock protein family B (small) member 8
IGF1R	insulin like growth factor 1 receptor
IGFBP4	insulin like growth factor binding protein 4
IL17RB	interleukin 17 receptor B
IL6ST	interleukin 6 signal transducer
INHBB	inhibin subunit beta B
INPP5F	inositol polyphosphate-5-phosphatase F
ISG20L2	interferon stimulated exonuclease gene 20 like 2
ITPK1	inositol-tetrakisphosphate 1-kinase
JAK2	Janus kinase 2
KAZN	kazrin, periplakin interacting protein
KCNK15	potassium two pore domain channel subfamily K member 15
KCNK5	potassium two pore domain channel subfamily K member 5
KDM4B	lysine demethylase 4B
KLF10	Kruppel like factor 10
KLF4	Kruppel like factor 4
KLK10	kallikrein related peptidase 10
KRT13	keratin 13
KRT15	keratin 15
KRT18	keratin 18

KRT19	keratin 19
KRT8	keratin 8
LAD1	ladinin 1
LRIG1	leucine rich repeats and immunoglobulin like domains 1
MAPT	microtubule associated protein tau
MAST4	microtubule associated serine/threonine kinase family member 4
MED13L	mediator complex subunit 13L
MED24	mediator complex subunit 24
MICB	MHC class I polypeptide-related sequence B
MLPH	melanophilin
MPPED2	metallophosphoesterase domain containing 2
MREG	melanoregulin
MSMB	microseminoprotein beta
MUC1	mucin 1, cell surface associated
MYB	MYB proto-oncogene, transcription factor
MYBBP1A	MYB binding protein 1a
MYBL1	MYB proto-oncogene like 1
MYC	MYC proto-oncogene, bHLH transcription factor
MYOF	myoferlin
NADSYN1	NAD synthetase 1
NAV2	neuron navigator 2
NBL1	NBL1, DAN family BMP antagonist
NCOR2	nuclear receptor corepressor 2
NPY1R	neuropeptide Y receptor Y1
NRIP1	nuclear receptor interacting protein 1
NXT1	nuclear transport factor 2 like export factor 1
OLFM1	olfactomedin 1
OLFML3	olfactomedin like 3
OPN3	opsin 3
OVOL2	ovo like zinc finger 2
P2RY2	purinergic receptor P2Y2
PAPSS2	3'-phosphoadenosine 5'-phosphosulfate synthase 2
PDLIM3	PDZ and LIM domain 3
PDZK1	PDZ domain containing 1
PEX11A	peroxisomal biogenesis factor 11 alpha
PGR	progesterone receptor
PLA2G16	phospholipase A and acyltransferase 3
PMAIP1	phorbol-12-myristate-13-acetate-induced protein 1
PODXL	podocalyxin like
PPIF	peptidylprolyl isomerase F
PRSS23	serine protease 23
PTGES	prostaglandin E synthase
RAB17	RAB17, member RAS oncogene family
RAB31	RAB31, member RAS oncogene family
RAPGEFL1	Rap guanine nucleotide exchange factor like 1
RARA	retinoic acid receptor alpha
RASGRP1	RAS guanyl releasing protein 1
RBBP8	RB binding protein 8, endonuclease
REEP1	receptor accessory protein 1
RET	ret proto-oncogene
RHOBTB3	Rho related BTB domain containing 3

RHOD	ras homolog family member D
RPS6KA2	ribosomal protein S6 kinase A2
RRP12	ribosomal RNA processing 12 homolog
SCARB1	scavenger receptor class B member 1
SCNN1A	sodium channel epithelial 1 subunit alpha
SEC14L2	SEC14 like lipid binding 2
SEMA3B	semaphorin 3B
SFN	stratifin
SH3BP5	SH3 domain binding protein 5
SIAH2	siah E3 ubiquitin protein ligase 2
SLC16A1	solute carrier family 16 member 1
SLC19A2	solute carrier family 19 member 2
SLC1A1	solute carrier family 1 member 1
SLC1A4	solute carrier family 1 member 4
SLC22A5	solute carrier family 22 member 5
SLC24A3	solute carrier family 24 member 3
SLC26A2	solute carrier family 26 member 2
SLC27A2	solute carrier family 27 member 2
SLC2A1	solute carrier family 2 member 1
SLC37A1	solute carrier family 37 member 1
SLC39A6	solute carrier family 39 member 6
SLC7A2	solute carrier family 7 member 2
SLC7A5	solute carrier family 7 member 5
SLC9A3R1	SLC9A3 regulator 1
SNX24	sorting nexin 24
SOX3	SRY-box transcription factor 3
STC2	stanniocalcin 2
SULT2B1	sulfotransferase family 2B member 1
SVIL	supervillin
SYBU	syntabulin
SYNGR1	synaptogyrin 1
SYT12	synaptotagmin 12
TBC1D30	TBC1 domain family member 30
TFAP2C	transcription factor AP-2 gamma
TFF1	trefoil factor 1
TFF3	trefoil factor 3
TGIF2	TGFB induced factor homeobox 2
TGM2	transglutaminase 2
THSD4	thrombospondin type 1 domain containing 4
TIAM1	TIAM Rac1 associated GEF 1
TIPARP	TCDD inducible poly(ADP-ribose) polymerase
TJP3	tight junction protein 3
TMEM164	transmembrane protein 164
TMPRSS3	transmembrane serine protease 3
TOB1	transducer of ERBB2, 1
TPBG	trophoblast glycoprotein
TPD52L1	TPD52 like 1
TSKU	tsukushi, small leucine rich proteoglycan
TTC39A	tetratricopeptide repeat domain 39A
TUBB2B	tubulin beta 2B class Iib
UGCG	UDP-glucose ceramide glucosyltransferase
UNC119	unc-119 lipid binding chaperone

WFS1	wolframin ER transmembrane glycoprotein
WISP2	cellular communication network factor 5
WWC1	WW and C2 domain containing 1
XBP1	X-box binding protein 1
ZNF185	zinc finger protein 185 with LIM domain

Table S2. Clinical and pathological features of each subtype in the TCGA cohort.

**TCGA (*n* = 1069)**	**Subtype**			
	ER+/HER2− (581)	HER2+ (176)	TNBC (159)	Unknown (153)
**Age**				
Median	59	59	55	
IQR	49–68	49–69	48–62	
**Race**				
White	430	109	90	
Black	72	25	54	
Asian	25	14	8	
Unknown	54	28	8	
**AJCC Stage**				
I	114	18	28	
II	314	106	102	
III	134	47	24	
IV	9	3	2	
Unknown	9	2	3	
**Grade**				
1	65	5	1	
2	200	39	10	
3	80	47	96	
Unknown	236	85	52	

Table S3. Comparison of clinical and pathological features between low and high estrogen response early score with ER-positive/HER2-negative breast cancer in the TCGA cohort.

ER+/HER2−	Estrogen Response Early Score		
	Low (389)	High (192)	*p*-value
**Age**			
Median	60	58	0.170
IQR	50–68	48–66	
**Race**			0.328
White	284	146	
Black	53	19	
Asian	15	10	
Unknown	37	17	
**T-category**			0.136
T1	100	66	
T2	215	100	
T3	62	22	
T4	11	4	
Unknown	1	0	
**N-category**			0.327
N−	173	94	
N+	210	95	
Unknown	6	3	
**M-category**			0.225
M−	309	165	
M+	7	3	
Unknown	73	24	
**AJCC stage**			0.049
Stage I	65	49	
Stage II	213	102	
Stage III	98	36	
Stage IV	7	2	
Unknown	6	3	

Table S4. Clinical and pathological features of each subtype in the METABRIC cohort.

**METABRIC (*n* = 1904)**	**Subtype**			
	ER+/HER2− (1355)	HER2+ (236)	TNBC (298)	Unknown (15)
**Age**				
Median	64	56	56	
IQR	54–72	51–65	45–66	
**AJCC Stage**				
0	2	2	0	
I	363	47	62	
II	568	96	130	
III	62	27	25	
IV	8	1	0	
Unknown	352	63	81	
**Nottingham grade**				
1	159	3	3	
2	652	50	36	
3	484	177	257	
Unknown	61	6	2	

Table S5. Comparison of clinical and pathological features between low and high estrogen response early score with ER-positive/HER2-negative breast cancer in the METABRIC cohort.

**ER+/HER2−**	**Estrogen Response Early Score**		
	Low (908)	High (447)	*p*-value
**Age**	64	63	<0.001
IQR	56–72	50–72	
**AJCC stage**			0.044
0	2	0	
I	228	135	
II	381	187	
III	48	14	
IV	3	5	
Unknown	246	106	
**Nottingham grade**			<0.001
1	106	53	
2	399	252	
3	363	121	
Unknown	40	21	

Table S6. Clinical and pathological features of each subtype in the GSE96058 cohort.

**GSE96058 (*n* = 3273)**	**Subtype**			
	ER+/HER2− (2425)	HER2+ (420)	TNBC (143)	Unknown (285)
**Age**				
Median	65	60	61	
IQR	54–72	49–69	51–72	
**Nottingham grade**				
1	470	4	2	
2	1327	102	20	
3	601	299	119	
Unknown	27	15	2	

Table S7. Comparison of clinical and pathological features between low and high estrogen response early score with ER-positive/HER2-negative breast cancer in the GSE96058 cohort.

**ER+/HER2−**	**Estrogen Response Early Score**		
	Low (1625)	High (800)	*p*-value
**Age**			
Median	65	64	<0.001
IQR	57–73	50–70	
**T-category**			0.71
T0	230	116	
T1	862	453	
T2	450	206	
T3	30	10	
T4	8	2	
Unknown	45	13	
**N-category**			1.00
N−	1385	689	
N+	198	98	
Unknown	42	13	
**M-category**			1.00
M−	1589	791	
M+	1	0	
	35	9	
**Nottingham grade**			<0.001
1	300	170	
2	851	476	
3	455	146	
Unknown	19	8	

Table S8. Comparison of metastatic regions between low and high estrogen response early score in the GSE124647 cohort.

**GSE124647 (*n* = 140)**	**Estrogen Response Early Score**		
	Low (94)	High (46)	*p*-value
**Metastatic regions**			0.204
Breast	9	10	
Liver	13	3	
Lymph node	31	13	
Bone	9	2	
Others	32	18	

Table S9. Clinical and pathological status of samples in the GSE20181 cohort.

Status	GSE20181 (*n* = 176)
**Response to treatment**	
R	110
NR	44
unknown	22
**Pre/post-treatment**	
Pre	58
Post (10–14 days)	58
Post (90 days)	60

Table S10. Clinical and pathological status of samples in the GSE33658 cohort.

Status	GSE33658 (*n* = 22)
**Response to treatment**	
R	10
NR	12
Pre	11
Post	11
**Subtype**	
ER+/HER2−	22
PR+	18
PR−	4

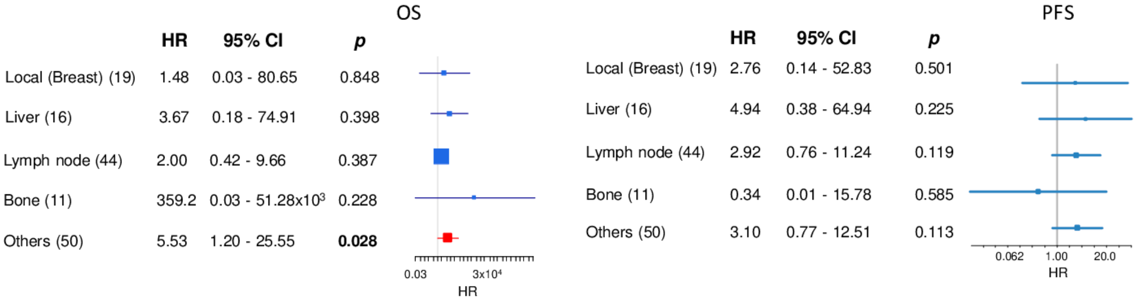

Figure S1. Association of the estrogen response early score with patient survival in several metastatic tumor in the GSE124647 cohort (n = 140). Forrest plots of the estrogen response early score with OS and PFS in each metastatic regions, including local (Breast (n = 19)), liver (n = 16), lymph node (n = 44), bone (n = 11), and others (n = 50). *P*-value was calculated by cox regulation model. HR; hazard ratio, CI; confidence interval.
